# Root resorption during orthodontic treatment with self-ligating or conventional brackets: a systematic review and meta-analysis

**DOI:** 10.1186/s12903-016-0320-y

**Published:** 2016-11-21

**Authors:** Jianru Yi, Meile Li, Yu Li, Xiaobing Li, Zhihe Zhao

**Affiliations:** 1 Department of Orthodontics, State Key Laboratory of Oral Diseases, West China Hospital of Stomatology, Sichuan University, #14, 3rd Section, South Renmin Road, Chengdu, 610041 People’s Republic of China; 2 Department of Pediatric Dentistry, State Key Laboratory of Oral Diseases, West China Hospital of Stomatology, Sichuan University, Chengdu, China

**Keywords:** Self-ligating bracket, Conventional bracket, Root resorption, Systematic review

## Abstract

**Background:**

The aim of this study was to compare the external apical root resorption (EARR) in patients receiving fixed orthodontic treatment with self-ligating or conventional brackets.

**Methods:**

Studies comparing the EARR between orthodontic patients using self-ligating or conventional brackets were identified through electronic search in databases including CENTRAL, PubMed, EMBASE, China National Knowledge Infrastructure (CNKI) and SIGLE, and manual search in relevant journals and reference lists of the included studies until Apr 2016. The extraction of data and risk of bias evaluation were conducted by two investigators independently. The original outcome underwent statistical pooling by using Review Manager 5.

**Results:**

Seven studies were included in the systematic review, out of which, five studies were statistically pooled in meta-analysis. The value of EARR of maxillary central incisors in the self-ligating bracket group was significantly lower than that in the conventional bracket group (SMD −0.31; 95% CI: −0.60–−0.01). No significant differences in other incisors were observed between self−ligating and conventional brackets.

**Conclusions:**

Current evidences suggest self-ligating brackets do not outperform conventional brackets in reducing the EARR in maxillary lateral incisors, mandible central incisors and mandible lateral incisors. However, self-ligating brackets appear to have an advantage in protecting maxillary central incisor from EARR, which still needs to be confirmed by more high-quality studies.

## Background

Currently, orthodontic treatment requires an average duration of 2–3 years. The lengthy treatment poses higher risks of numerous side effects to patients, among which external apical root resorption (EARR) has been frequently reported [1].

EARR could be defined as the blunting and shortening of root apex caused by the pathologic loss of the cementum and dentine. It is widely accepted that the elimination of hyalinization zone is critical for the physiological tooth movement [2]. However, this process is initiated by microphage-like cells from periodontal ligament blood supply, which could also damage the nearby cementoblast layer covering the cementoid [3]. After the exposure of cementum, the denuded root surface is more susceptible to resorption by scavenger cells and osteoclasts during the hyaline tissue elimination [4].

The EARR induced by orthodontic treatment, most of which were graded as minor or moderate, generally resulted in no significantly clinical symptoms [5]. Nevertheless, the alternations of root length could cause an unfavorable crown-root ratio, which should be cared with caution when patients suffered from alveolar bone loss simultaneously [6]. Therefore, this issue deserves more attention since an increasing number of adults who are more vulnerable to periodontal diseases are seeking for orthodontic treatment.

Although EARR in orthodontics has been considered as an iatrogenic problem, the relationship between orthodontic treatment-related factors and EARR has never been fully answered. Previous study suggested the occurrence of EARR appears to be closely associated with mechanical factors during orthodontic treatment [6]. The treatment duration and force magnitudes have been claimed as contributory factors to the development of EARR [7]. Moreover, the distance of tooth movement, type of force application and treatment techniques have also been reported to influence the incidence and severity of EARR during orthodontic treatment [8].

In current orthodontic clinics, self-ligating (SL) brackets have been widely used due to the free of ligature by additional rubber elastics or steel ligatures, and the resulting reduction of the chair time and the frictional resistance between archwire and brackets [9]. Furthermore, SL brackets have been claimed to have advantages in shorter treatment duration, less orthodontic pain and better oral hygiene [10]. The popularization of SL brackets raises the question that whether they would have different effect on EARR compared with conventional brackets (non-SL brackets). Thus a critical systematic review would be quite beneficial for clinicians. In present study, we carried out a systematic review to comprehensively evaluate, in an evidence-based way, the EARR in fixed orthodontic treatment with SL or non-SL brackets.

## Methods

This systematic review and meta-analysis was performed referring to Cochrane Handbook for systematic Reviews of Interventions and Perferred Reporting Items for Systematic Reviews and Meta-Analyses (PRISMA) checklist [11]. Two reviewers (J.Y and M.L) independently conducted the study inclusion, data extraction and assessing the risk of bias of the retrieved articles. Cohen’s Kappa coefficient was calculated to measure agreement in selecting studies [12], the score of 0.88 suggests the inter-examiner bias was low [13]. Any discrepancy in the conduction of this systematic review was settled by discussing with a third reviewer (Y.L).

### Search strategy

An extensive electronic search was conducted through databases including Cochrane Central Register of Controlled Trials (CENTRAL), PubMed, EMBASE, China National Knowledge Infrastructure (CNKI) and SIGLE. The search strategy combined MeSH heading words with free text words without context. The adopted MeSH terms were ‘root resorption’ and ‘orthodontic appliances’. The free text words included root shortening, root alternation, self-ligating and ligating. The search strategies for databases were established based on that for PubMed. The electronic search was conducted on Apr 10, 2016. In addition, manual search was undertaken among issues of relevant journals and the reference lists of retrieved articles by reviewing the titles and abstracts. No language restriction was applied during the literature search.

### Criteria for included studies

The inclusion criteria were as follows: (1) Types of studies: randomized controlled trials (RCTs), controlled clinical trials (CCTs) and cohort studies are eligible; (2) Types of participants: subjects should be healthy patients who required fixed orthodontic treatment; (3) Types of Intervention: patients received fixed orthodontic treatment using SL or non-SL brackets; (4) Outcomes: the reduction of root length (in millimeters and in percentage).

The exclusion criteria were as follows: (1) Review articles, descriptive studies, opinion articles, case reports or abstracts; (2) Animal studies; (3) Studies involving subjects with systemic diseases or diagnosed as root absorption before orthodontic treatment.

### Data extraction and analysis

A customized data extraction form was developed. Relevant information regarding study designs, participant information, interventions, outcomes (EARR, evaluated teeth, measurement approach) and treatment duration were extracted.

### Risk of bias evaluation

The risk of bias assessment form recommended by Saltaji et al. [14] and Wu et al. [15] was used to evaluate the risk of bias of included studies. This assessment system evaluated the risk of bias on the basis of four broad perspectives: study design (8 items), study measurements (3 items), statistical analysis (3 items) and baseline information (1 item), with a maximum score of 19. the item was scored as 1 point (√) when the trial reported the domain properly, as 0.5 point (≠) when the trial partially fulfilled the criteria and as 0 point (×) when the trial did not fulfill the methodological criteria (Table 1). The study was assessed as ‘low risk of bias’ when the score was higher than 15, ‘moderate risk of bias’ when the score was between 10 and 15, and ‘high risk of bias’ when the score was less than 10 points [14, 15].

**Table 1:** Risk of bias assessment form for the recruited studies

Study Design (11√)	
1. Objective – clearly defined (√)	
2. Population – adequately described (√)	
3. Sample size – considered adequate (√)	
4. Selection criteria – clearly described (√), adequate (√)	
5. Randomization or consecutive selection – stated (√)	
6. Follow-up length – clearly described (√)	
7. Timing – prospective design (√)	
8. Type of Study – RCT (3√), CCT (2√), Cohort study (√)	
Study measurements (3√)	
9. Measurement method – appropriate (√)	
10. Blinding – stated (√)	
11. Reliability – Described (√)	
Statistical Analysis (4√)	
12. Dropouts – accounted (√)	
13. Statistical analysis – appropriate (√)	
14. Presentation of data – exact P value (√), variability measures (SD or CI) stated (√)	
Baseline (1√)	
15. Datum line situation: − two groups were calibrated and most consistent (√)	

Maximum score = 19

### Statistical analysis

Review Manager (RevMan5.3; Nordic Cochrane Centre, Cochrane collaboration, Copenhagen, Denmark) was used for the meta-analysis of quantitative data. For continuous data, the standardized mean difference (SMD) and its 95% confidence interval (CI) were adopted as the treatment effects and pooled for analysis. The heterogeneity of recruited studies was explored by I^2^ statistic. The random-effect model would be adopted for the meta-analysis if the heterogeneity was assessed as high (I^2^ > 50%); Otherwise, the fixed-effect model was employed. The statistical significance of the hypothesis test was set as *p* < 0.05 (2-tailed Z test). Subgroup analysis was performed on the basis of treatment durations. Sensitivity analysis was performed to test the stability of overall results in the meta-analysis by omitting study separately. Descriptive analysis was adopted when the statistical synthesis of data failed. The funnel plot and Beggs’ rank correlation test were used to determine the publication bias if the number of recruited studies exceeded 10 [16].

## Results

### Search results

**Fig. 1:**
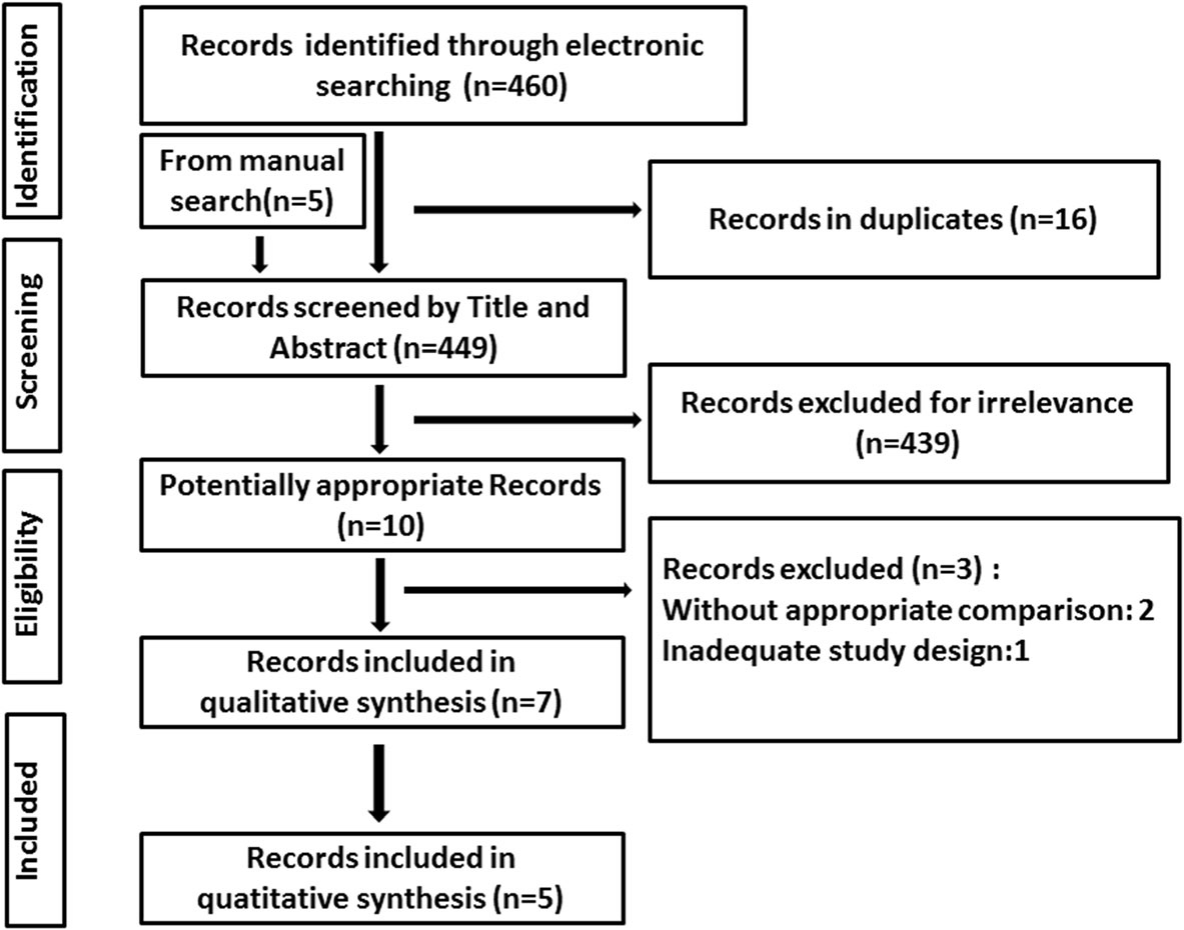
Flow diagram of the study inclusion of the systematic review and meta-analysis

A total of 449 articles were retrieved through the electronic and manual searching. Of these, 439 irrelevant citations were excluded and ten studies were considered potentially eligible after evaluating titles and abstracts. The full-texts of the reserved ten studies were retrieved and assessed referring to the inclusion and exclusion criteria. Finally, seven studies [17–23] fulfilled the criteria and were included in present systematic review, five of which [17–21] were pooled for meta-analysis. A flow diagram of the study selection process was demonstrated in Fig. 1.

### Characteristics of included studies

A total of 553 participants were included. All the participants were systemically healthy and received fixed orthodontic treatment. Among the seven recruited studies, one was categorized as RCT, two were CCTs and the other four studies were cohort studies (Table 2). One of the included studies was published in Chinese [18], and the remaining six were in English. 1 RCT, 2 CCTs and two cohort studies were enrolled in the meta-analysis. The detailed information of included studies regarding study design, participants, interventions, outcome and measurements were summarized in Table 2.

**Table 2:** General information of recruited studies

Study	Study design	Participants	Comparisons	Outcomes (Method)	Evaluated teeth	treatment duration
Blake et al. (1995) [20]	CCT	S:n = 30(M12,F18;12.8 ± 2.3y) N:n = 33(M16,F17;13 ± 2.5y)	SL bracket (Speed, Strite inductries) vs non-SL bracket	Root resorption in percentage (periapical radiograph)	(11,21),(12,22),(13,23),(14,24)	S:20.9 ± 4.36 month N:20.6 ± 4.6 month
Scott et al. (2008) [21]	RCT	S:n = 32 (M12, F20;16.19 ± 3.68y) N:n = 28 (M19, F9; 16.38 ± 5.28y)	SL brackets vs non-SL bracket (Synthesis, Ormco)	Root resorption in millimeter (periapical radiograph)	Mandibular right central incisor	S:8.5 ± 2.1 month N:8.1 ± 2.7 month
Pandis et al. (2008) [22]	Cohort study	S:n = 48(M17,F31;13.29 ± 1.57y) N:n = 48(M12,F36;13.14 ± 1.73y)	SL bracket (Damon2, Ormco) vs Non-SL bracket (Microarch, GAC)	Root resorption in millimeter (panoramic radiographs)	Maxillary incisors	S:26.89 ± 5.94 month N:25.97 ± 6.65 month
Leite et al. (2012) [17]	CCT	n = 19(20.6y,min11,max30) S:n = 11(M6,F5) L:n = 8(M2,F6)	SL bracket (EasyClip) vs non-SL bracket (3 M)	Root resorption in millimeter (CBCT)	(11,21),(12,22),(13,23),(14,24)	6 month
Liu et al. (2012) [18]	Cohort study	S:n = 35(M7,F8;15.13y) L:n = 35(M9,F6;14.93y)	SL bracket (Damon3, Ormco) vs non-SL bracket	Root resorption in millimeter (periapical radiographs)	(11,21),(12,22),(13,23),(14,24)	S:20.4 ± 5.04 month N:16.8 ± 2.66 month
Jacobs et al. (2014) [23]	Cohort study	S:n = 139(M56,F83;12.6 ± 2.3y) L:n = 74(M23,F51;12.1 ± 2.2y)	SL bracket (SmartClip 3 M) vs non-SL bracket (Victory,3 M)	Root resorption in percentage (panoramic radiographs)	Maxillary and mandibular incisors	S:20.7 ± 4.9 month N:18.1 ± 5.3 month
Chen et al. (2015) [19]	Cohort study	S:n = 35(M17,F18;13.52 ± 2.84y) L:n = 35(M16,F19;13.42 ± 2.50y)	SL bracket (Damon3, Ormco) vs Non-SL bracket (3 M)	Root resorption in millimeter (periapical radiographs)	(11,21),(12,22),(13,23),(14,24)	S:20.53 ± 3.62 month N:20.34 ± 3.40 month

### Risk of bias of the included studies

The detailed information regarding the risk of bias evaluation was summarized in Table 3. Out of the seven included studies, one study had a low risk of bias and the other six studies had a moderate risk of bias. The five studies underwent statistical pooling included one study with low risk of bias and four studies with moderate risk of bias. All recruited studies had a clearly defined objective, calibrated baseline information, effective follow-up duration, appropriate study design, measurements and statistical analysis.

**Table 3:** Risk of bias evaluation of included studies^a^

Study ID	Study design								Study measurements			Statistical analysis			Baseline	Total
	1	2	3	4	5	6	7	8	9	10	11	12	13	14	15	
Blake et al. (1995) [20]	√	√	≠	××	×	√	√	2√	√	×	≠	×	√	×√	√	11
Scott et al. (2008) [21]	√	√	≠	√×	√	√	√	3√	√	≠	√	√	√	×√	√	15.5
Pandis et al. (2008) [22]	√	√	≠	√√	×	√	×	√	≠	×	≠	×	√	√≠	√	11
Leite et al. (2012) [17]	√	√	×	√√	×	√	√	2√	√	×	×	×	√	√√	√	13
Liu et al. (2012) [18]	√	√	×	√×	×	√	×	√	√	×	≠	×	√	√√	√	10.5
Jacobs et al. (2014) [23]	√	≠	≠	√√	×	√	×	√	√	×	×	×	√	×√	√	10
Chen et al. (2015) [19]	√	√	≠	√√	×	√	×	√	√	×	×	×	√	×√	√	10.5

^a^1 to 15: methodologic criteria in Table 1

√ = 1point; ≠ = 0.5point; × = 0point

### External apical root resorption

**Fig. 2:**
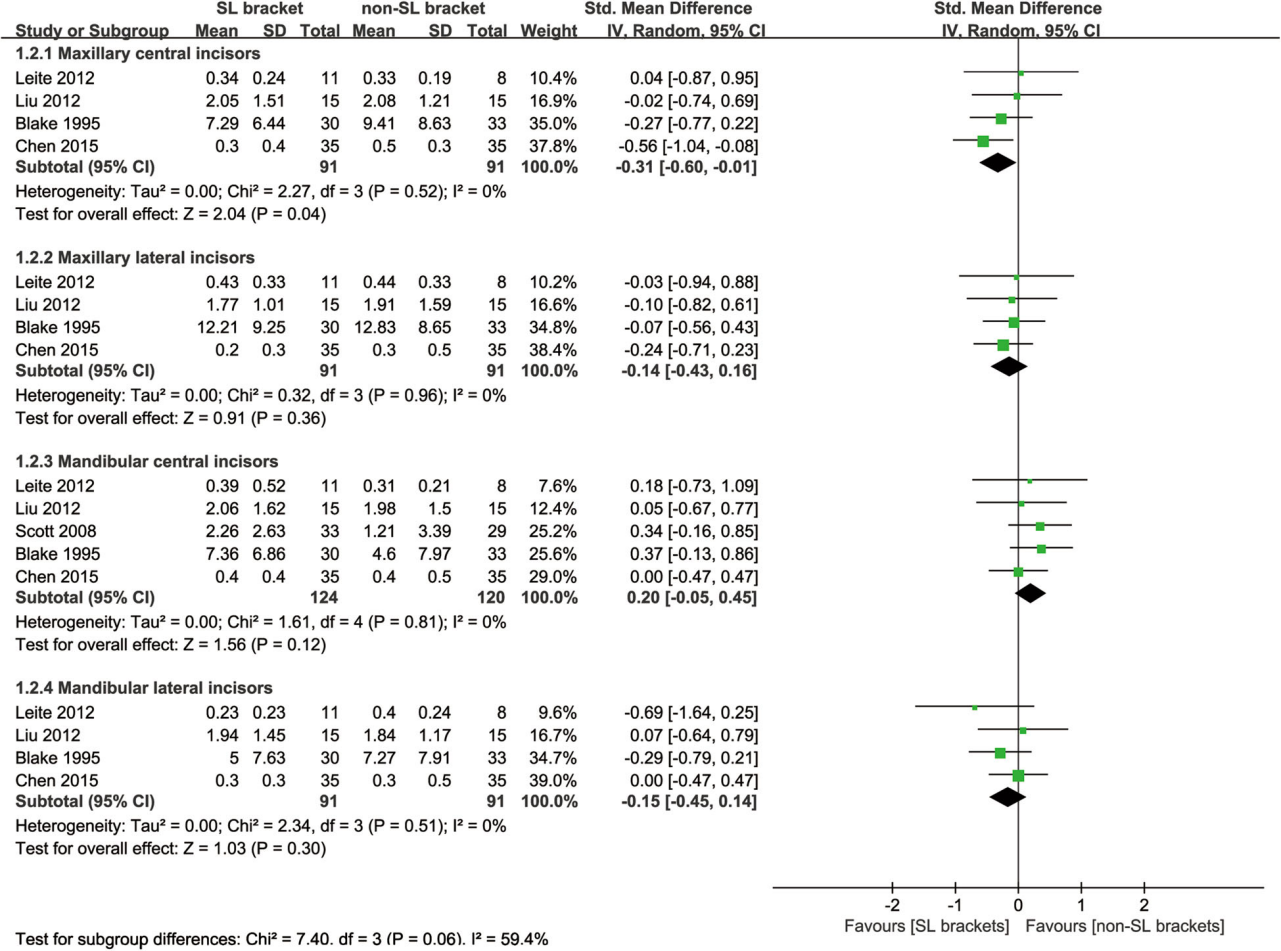
Meta-analysis of EARR values comparing SL with non-SL brackets

Five studies reported the values of EARR with teeth grouped as maxillary central incisors, maxillary lateral incisors, mandibular central incisors and mandibular lateral incisors during fixed orthodontic treatment. The feasible data was statistically pooled to compare the values of EARR between two types of brackets. The meta-analysis results showed that the patients using SL brackets suffered less EARR of maxillary central incisor compared to those using non-SL brackets (SMD −0.31; 95% CI:−0.60–−0.01) (Fig. 2), while no significant difference was detected in maxillary lateral incisors (SMD −0.14; 95% CI:−0.43–0.16) (Fig. 2), mandibular central incisors (SMD 0.20; 95% CI: −0.05–0.45) (Fig. 2) and mandibular lateral incisors (SMD −0.15; 95% CI: −0.45–0.14) (Fig. 2).

Out of the five included studies in meta-analysis, three studies [18–20] had follow-ups of approximate 20 months while the other two studies [17, 21] had comparatively shorter treatment durations (Table 2). The subgroup analysis on the basis of treatment durations was conducted to decrease the potential heterogeneity, showing no difference in either studies classified as long-term studies or those short-term studies, except that no difference in the EARR of maxillary central incisors between two types of brackets was observed in those short-term studies (Table 4).

**Table 4:** Subgroup analysis data summary

Evaluated teeth	Long-term studies			Short-term studies
	SMD	95% confidence interval	SMD	95% confidence interval
Maxillary CI^a^	−0.35	−0.66–−0.04	0.04	−0.87–0.95
Maxillary LI^b^	−0.15	−0.46–0.16	−0.03	−0.94–0.88
Mandibular CI	0.15	−0.16–0.46	0.31	−0.13–0.75
Mandibular LI	−0.10	−0.40–0.21	−0.69	−1.64–0.25

^a^indicates central incisors

^b^indicates lateral incisors

The other two studies [22, 23] were not included in the meta-analysis due to the lack of comparability of data. Pandis et al. [22] found the EARR of maxillary incisors were not associated with different brackets using a multivariate model (coefficient: 0.37; SE: 0.20; *p* =0.06). Jacobs et al. [23] also reported no significant difference in EARR of incisors between SL or non-SL brackets (3.0 ± 5.6% vs 4.5 ± 6.6%, *p* > 0.05).

### Sensitivity analysis

Leite et al. [17] evaluated the EARR using CBCT while the other four studies in meta-analysis adopted periapical radiographs. Moreover, the participants in Scott et al. and Leite et al. [17, 21] are featured with comparatively heterogenous age. Therefore, the sensitivity analysis was conducted by omitting the two studies separately. The exclusion of Leite et al. and Scott et al. resulted in no changes in the overall results in all evaluated teeth (Table 5).

**Table 5:** Sensitivity analysis data summary

	Maxillary CI^a^	Maxillary LI^b^	Mandibular CI	Mandibular LI
Exclusion of Scott et al.	−0.31(−0.60–0.01)	−0.14(−0.43–0.16)	0.15(−0.14–0.44)	−0.15(−0.45–0.14)
Exclusion of Leite et al.	−0.35(−0.66–0.04)	−0.15(−0.46–0.16)	0.20(−0.06–0.46)	−0.10(−0.40–0.21)

^a^indicates central incisors

^b^indicates lateral incisors

## Discussion

This systematic review was performed to provide data on the EARR during orthodontic treatment using SL or non-SL brackets. After a comprehensive literature search and evaluation, seven articles were recruited in this systematic review, among which, five studies were statistically pooled for the quantitative analysis. The meta-analysis results suggest that SL bracket is superior to non-SL bracket in protecting maxillary central incisors from EARR (Fig. 2). While no significant differences in the EARR of maxillary lateral incisors, mandibular central incisors and mandibular lateral incisors were found between two types of brackets (Fig. 2). The results based on currently available evidences may suggest the priority of using SL brackets when patients with more vulnerable maxillary central incisors or diminished root-crown ratio are receiving orthodontic treatment.

The sensitivity analysis omitting Leite et al. and Scott et al. [17, 21] brought about no changes to the overall effects in all evaluated teeth (Table 5). The consistent outcomes seemed to be the indicative of the robustness of the meta-analysis results. Nevertheless, only five studies were included in the quantitative analysis. Moreover, it should be noted the upper limit of the SMD (95% CI) in the meta-analysis comparing the occurrence of EARR in maxillary central incisors was close to 0 (Fig. 2, Table 5). Thus the aforementioned advantage of SL brackets is recommended to be interpreted cautiously in clinical settings.

The exact mechanism of the EARR development is still unclear, but it is generally accepted the root resorption is positively associated with force magnitudes and apical movement distance [6, 24]. Recent systematic review suggests that SL brackets have no superiority in treatment efficiency [9]. Nevertheless, owing to the free of ligation by steel ligatures and rubber elastics, archwire could have more free space in slots of SL brackets than in non-SL brackets, which could result in the lower frictional force in SL bracket systems and might exert smaller force to teeth in the initial alignment [25, 26]. On the other hand, in typical cases characterized by maxillary protrusion, the root of maxillary central incisors would move labially in the initial alignment stage and then move palatally during the space closure. The reciprocating and distant movement could cause the high incidence of EARR in maxillary central incisors [24]. Taken together, maxillary central incisors is under a higher risk of EARR development, thus the lower force magnitude transmitted to teeth in SL systems could be more readily to produce a significant protective effect from tooth resorption in maxillary central incisors rather than other evaluated teeth (Fig. 2). Anyway, this opinion is mostly empirical and needs to be further identified.

The treatment duration has been suggested as a risk factor to the development of root resorption [6]. Among the five studies in meta-analysis, Leite et al. and Scott et al. [17, 21] had shorter follow-up duration (about 6 months) and were thus considered as the short-term studies, while the other three studies were classified as long-term studies due to the follow-ups of approximate 20 months (Table 2). In the subgroup analysis, the protective effect of SL brackets on maxillary central incisors is significant to the long-term studies, while not to the short-term study (Table 4), indicating the protective effect of SL brackets seem to be valid only in a long run. Nevertheless, more studies are needed to obtain a more reliable result.

Previous studies demonstrated the association between EARR occurrence and numerous mechanical factors including force magnitude, amount of tooth movement, force type and treatment appliance [6, 7]. The heterogeneity of these factors could influence the results concerning root resorptions. However, limited information regarding aforementioned factors is available in included studies of this review, which could reduce the stability of our results and should be considered in future clinical trials.

The diagnosis of EARR has been mainly through radiographs. Out of the seven included studies, four studies adopted periapical radiographs, two used panoramic radiographs, and the other one employed CBCT (Table 2). Though no significant difference was detected in the sensitivity analysis that excludes the study using different radiographic tool (Table 5), the varied magnifications and distortions of the foregoing techniques could restrict the comparability of recruited studies and affect the overall results [27]. The recent study has suggested CBCT as a more reliable and valid measurement for root resorption compared to the 2-dimensional approaches including periapical films and panoramic radiographs since it enables clinicians to visualize and evaluate the root resorption on any surface of roots and eliminates the structure superimposition [28]. From the perspective of measurement accuracy and better comparability, future studies should use CBCT to assess the occurrence of EARR in orthodontic treatment. However, the higher radiation exposure and more expenses of CBCT should also be considered [29].

### Limitations

Firstly, though extensive literature search was conducted, only five studies were included in the meta-analysis, leading to the deficient statistical power. Secondly, out of the five studies in meta-analysis, one study evaluated the percentage of root reduction while the other four reported the absolute values of root resorption. Though SMD was computed in the quantitative analysis, the outcomes should be interpreted with caution in clinical settings. Thirdly, though subgroup and sensitivity analysis was performed, the source of heterogeneity might not be thoroughly investigated since no information regarding several risk factors of EARR, like tooth movement distance and force magnitude, was reported in primary studies. Fourthly, funnel plots for publication bias assessment has not been conducted because only five studies were included in the meta-analysis. Therefore, further high-quality original studies are needed to arrive at a more stable conclusion.

## Conclusion

Based on present limited evidence, SL brackets appear to have a long-term protective effect to maxillary central incisors from root resorption compared to non-SL brackets. No different influences on other incisors were detected. The results of this study could suggest the priority of SL brackets to patients with more susceptible maxillary central incisor roots or unfavorable crown-root ratio in fixed orthodontic treatment. However, methodologically sound clinical trials are required to provide more reliable evidences regarding this issue.

## Abbreviations

EARR: External apical root resorption
non-SL bracket: Conventional brackets
SL brackets: Self-ligating bracket
SMD: Standardized mean difference
